# UNFULFILLED PROMISES OF WORKING IN LATER LIFE OF NEW ZEALAND WOMEN

**DOI:** 10.1093/geroni/igz038.464

**Published:** 2019-11-08

**Authors:** Christine G Unson

**Affiliations:** Southern Connecticut State University, New Haven, Connecticut, United States

## Abstract

This critical study on women and work-life extension compared motivations, needs satisfaction, barriers and resources faced by ethnically diverse women in high and low status occupations. Twenty-seven women (mean age = 64; age range 55 to 79 years) who at 55 years or older, had changed careers or places of employment, or who had retired and currently volunteer were interviewed. Four were Asian/Pacific Islanders, seven Māori and 16 European New Zealanders. Themes were compared between women in low and high status occupations. Instrumental motivations were most common among women in lower-status occupations and entrepreneurs whereas idealistic motivations were more prevalent with higher- status occupations and volunteer posts. Themes related to needs satisfaction show that work met basic and security needs among lower-status occupations but self-actualization needs were expressed more frequently in higher-status occupation. Feelings of belonging and a sense of achievement were cited by most participants. Themes relating to barriers faced by participants in lower-status occupations and by minority women include inadequate skills and access to information, and racial, ageist, and sexist discrimination. Themes also revealed multiple sources of supports: employers provided training and managerial support, and for some non-profits, a collaborative working atmosphere and co-workers who met older workers’ needs for belonging and sense of achievement, and who potentially, as a unit, could advocate for better working conditions for older workers. Work-life extension has the potential for creating classes of older female workers with differential access to work opportunities and level of aspirations achievable.

